# Retraction: Molecular and cellular mechanisms of cigarette smoke-induced myocardial injury: Prevention by Vitamin C

**DOI:** 10.1371/journal.pone.0340142

**Published:** 2026-01-06

**Authors:** The PLOS One Editors

After this article [1] was published, concerns were raised regarding results presented in Figs 2, 4 and 6.

Specifically:

- In the following western blot panels, the marker “M” lanes appear similar: Fig 2A, Fig 2B, Fig 6B, Fig 6C in [1], and Fig 4A and Fig 4B in [2], retracted in [3]
- The following panels appear to partially overlap:
  - Fig 2C CS 2 Weeks 15mg vit C DAPI and Fig 2C 6 Weeks CS 15mg vit C DAPI
  - Fig 4E AIR 8 Weeks 0.5mg vit C TUNEL and Fig 4E AIR 8 Weeks 15mg vit C TUNEL

Regarding the marker lanes in [1] and [2], the first author stated these lanes are similar because the experiments were run under similar percentage SDS-PAGE and the same voltage, and the protein marker is shown for reference only. They provided data underlying Figs 2A, 2B, 6B, and 6C, however editorial review raised additional concerns about the integrity of the underlying data provided.

Regarding Fig 2C, the first author disagreed that these panels appear similar. Regarding Fig 4E, they agreed with the concerns identified for the AIR 8 Weeks 0.5 mg vit C TUNEL and Fig 4E AIR 8 Weeks 15 mg vit C TUNEL panels. The first author stated these panels should be different. The underlying image data provided did not resolve these concerns and instead raised further concerns about the overall data handling.

In light of the above concerns that question the reliability of the reliability of the reported results, the *PLOS One* Editors retract this article.

AD did not agree with the retraction. ND, AG, SD, and DJC either did not respond directly or could not be reached. IBC is deceased.
